# Supplementary material for: An inner activation gate controls TMEM16F phospholipid scrambling
Source: Nat Commun. 2019 Apr 23;10:1846. doi: 10.1038/s41467-019-09778-7 (PMC6478717; doi:10.1038/s41467-019-09778-7)
Supplement: Supplementary file 1 — Supplementary Information [file 41467_2019_9778_MOESM1_ESM.pdf]

Supplementary Information for

An inner activation gate controls TMEM16F phospholipid scrambling

Trieu Le, Zhiguang Jia, Son C. Le, Yang Zhang, Jianhan Chen, Huanghe Yang*

This PDF file includes:

Supplementary Figs. 1-7
Supplementary Tables 1-2

Other Supplementary Materials for this manuscript includes the following:

Supplementary Movies 1-2
Source Data file

a

```
[cyan] Trans-membrane domain  [red] Gate
mTMEM16A  1     MRVPEKYSTLPAE-------DRSVHIVNICAIEDLGYLPSEGTLLNSLSV-DPDAECKYG----LYFRDGKRKVDYILVY  68
mTMEM16F  1     MQMMTRKVLLNMELEEDDDEDGDIVLENF---DQTIVCPTFGSLENQQDFRTPEFEEFNGKPDSLFFTDGQRRIDFILVY  77
nhTMEM16        --------------------------------------------------------------------------------

mTMEM16A  69    HHKRASGSRTLARRGLQNDMVLGTRSVRQDQPLPGKGSPVDAGSPEVPMDYHEDDKRFRREEYEGN-------LLEAGLE  141
mTMEM16F  78    EDESKKENNK-------------------------KGT-------------NEKQKR-KRQAYESN-------LICHGLQ  111
nhTMEM16  1     ------------------------MSNLKDFSQPGSGQESNFG---VDFVIHYKVPAAERDEAEAGFVQLIRALTTVGLA  53

mTMEM16A  142   LENDEDTKIHGVGFVKIHAPWHVLCREAEFLKLKMPTKKVYHISETR---GLLKTINSVLQKITDPIQPKVAEHRPQTTK  218
mTMEM16F  112   LEATRSVSDDKLVFVKVHAPWEVLCTYAEIMHIKLPLKP--NDLKTRSPFGNLNWFTKVLRVNESVIKPE--------QE  181
nhTMEM16  54    TEVRHGENESLLVFVKVASP-------------DLFAKQV-----YRARLGDWLHGVRVSAPHNDIAQAL----------  105

mTMEM16A  219   RLSYPFSREKQHLFDLTDRDSFFDSKTRSTIVYEILKRTTCTKAKYSM-------GITSLLANGVY-SAAYPLHDG--DY  288
mTMEM16F  182   FFTAPFEKSRMNDFYILDRDSFFNPATRSRIVYFILSRV-----KYQVMNNVNKFGINRLVSSGIY-KAAFPLHDCRFNY  255
nhTMEM16  106   ------------------QDEPVVEAERLRLIYLMITKP----------HNEGGAGVTPTNAKWKHVESIFPLHSHSFNK  157
                                                                 TM1
mTMEM16A  289   EGDNVEF-NDRKLLYEEWASYGVFYKYQPIDLVRKYFGEKVGLYFAWLGAYTQMLIPASIVGVIVFLYGCATVDENIPSM  367
mTMEM16F  256   ESEDISCPSERYLLYREWAHPRSIYKKQPLDLIRKYYGEKIGIYFAWLGYYTQMLLLAAVVGVACFLYGYLDQDNCTWSK  335
nhTMEM16  158   E------------WIKKWSSKYTL-EQTDIDNIRDKFGESVAFYFAFLRSYFRFLVIPSAFGFGAWLL------------  212
                                                                 TM2
MTMEM16A  368   EMCDQRYN--ITMCPLCDKTCSYWKMSSACATARASHLFDNPATVFFSVFMALWAATFMEHWKRKQMRLNYRWDLTGFEE  445
MTMEM16F  336   EVCDPDIGGQILMCPQCDRLCPFWRLNITCESSKKLCIFDSFGTLIFAVFMGVWVTLFLEFWKRRQAELEYEWDTVELQQ  415
nhTMEM16  213   ------LG---------------------------------QFSYLYALLCGLWSVVFFEYWKKQEVDLAVQWGVRGVSS  253
                                                                                  TM3
mTMEM16A  446   EEEAVKDHPRAEYEARVLEKSLRKESRNKETDKVKLTWRDRFPAYFTNLVSIIFMIAVTFAIVLGVIIYRISTAAALAM-  524
mTMEM16F  416   EEQA-----RPEYEAQCNHVVINEITQEEE--RIPFTTCGKCIRVTLCASAVFFWILLIIASVIGIIVYRLSVFIVFSTT  488
nhTMEM16  254   IQQS-----RPEFEW--------EHEAEDPITGEPVKVYPPMKRVKTQLLQIPFALACVVA--LGALIVTCNSLEVFINE  318
                                            TM4                                   TM5
mTMEM16A  525   -----NSSPSVRSNI--RVTVTATAVIINLVVIILLDEVYGCIARWLTKIEVPKTEKSFEERLTFKAFLLKFVNSYTPIF  597
mTMEM16F  489   LPKNPNGTDPIQKYLTPQMATSITASIISFIIIMILNTIYEKVAIMITNFELPRTQTDYENSLTMKMFLFQFVNYYSSCF  568
nhTMEM16  319   VY-----SGPGKQYL------GFLPTIFLVIGTPTISGVLMGAAEKLNAMENYATVDAHDAALIQKQFVLNFMTSYMALF  387
                                                                                   TM6
mTMEM16A  598   YVAFFKGRFVGRPGDYVYIFRSF-----------------RMEECAPGGCLMELCIQLSIIMLGKQLIQNNLFEIGIPKM  660
mTMEM16F  569   YIAFFKGKFVGYPGDPVYLLGKY-----------------RSEECDPGGCLLELTTQLTIIMGGKAIW-NNIQEVLLPWV  630
nhTMEM16  388   FTAFVYIPF----GHILHPFLNFWRATAQTLTFSEKELPTREFQINPA----RISNQMFYFTVTAQIV-NFATEVVVPYI  458

                                                              TM7                        TM8
mTMEM16A  661   KKFIRYLKLRRQSPSDREEYVKRKQRYEVDFNLE-P--FAGLTPEYMEMIIQFGFVTLFVASFPLAPLFALLNNIIEIRL  737
mTMEM16F  631   MNLIGRYKRVSGS-------EKITPRWEQDYHLQ-PMGKLGLFYEYLEMIIQFGFVTLFVASFPLAPLLALVNNILEIRV  702
nhTMEM16  459   KQQAFQKAKQLKSGSKVQEDHEEEAEFLQRVREECTLEEYDVSGDYREMVMQFGYVAMFSVAWPLAACCFLVNNWVELRS  538

                                          TM9
mTMEM16A  738   DAKKFVTELRRPVAIRAKDIGIWYNILRGVGKLAVIINAFVISFTSDFIPRLVYLYMYS-------QNGTMHGFVNHTLS  810
mTMEM16F  703   DAWKLTTQFRRMVPEKAQDIGAWQPIMQGIAILAVVTNAMIIAFTSDMIPRLVYYWSFSIPPYGDHTYYTMDGYINNTLS  782
nhTMEM16  539   DALKIAISSRRPIPWRTDSIGPWLTALSFLSWLGSITSSAIVYLCSNS--------------------------------  586
                                                                       TM10
mTMEM16A  811   SFNVSDFQNGTAPNDPLDLGYEVQICRYKDYREPPWSEHKYDISKDFWAVLAARLAFVIVFQNLVMFMSDFVDWVIPDIP  890
mTMEM16F  783   VFNITDFKNTDKENPYIGLG-NYTLCRYRDFRNPPGHPQEYKHNIYYWHVIAAKLAFIIVMEHIIYSVKFFISYAIPDVS  861
nhTMEM16  587   -------KNGTQGEASPLKA---------------------------WGL----LLSILFAEHFYLVVQLAVRFVLSKLD  628

mTMEM16A  891   KDISQQIHKEKVLMVELFMREEQGKQQLLDTWMEKEKPRDVPCNNHSPTTHPEAGDGSPVPSYEYHGDAL----------  960
mTMEM16F  862   KITKSKIKREKYLTQKL-LHESHLKDLTKNMGIIAERIGGTVDNSVRPKLE-----------------------------  911
nhTMEM16  629   SPGLQKERKERFQTKKRLLQE--------NLGQDAAEEAAAPGIEHSEKITREALE-EEARQASIRGHGTPEEMFWQRQR  699

mTMEM16A        ------------------------------------
mTMEM16F        ------------------------------------
nhTMEM16  700   GMQETIEIGRRMIEQQLAAGKNGKKSAPAVPSEKAS                                              735
```

b **c**

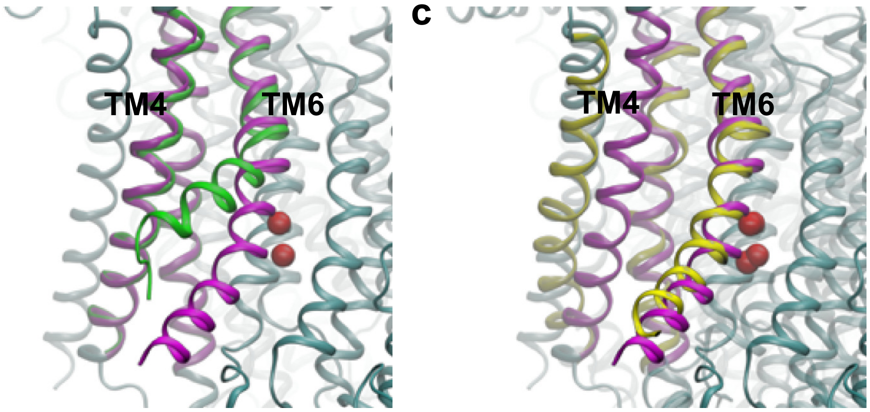

5OYB (Magenta) and 5OYG (Green) 5OYB (Magenta) and 4WIS (Yellow)

d

Transmembrane domain	Putative inner gate residues		
	nhTMEM16	TMEM16A	TMEM16F
TM4	V337	L543	F518
TM5	S382	S588	Y563
TM6	F440	I637	I612

Supplementary Figure 1. Comparison between the sequences and structures of TMEM16A, TMEM16F and nhTMEM16. a Sequence alignment of mTMEM16A, mTMEM16F and nhTMEM16. Transmembrane (TM) domains and the inner gate residues are highlighted in cyan and red respectively. **b** Superposition of Ca^{2+}-free (PDB: 5OYG; green) and Ca^{2+}-bound (PDB: 5OYB; magenta) mTMEM16A structures, showing the movement of TM 6 around the glycine hinge. **c** Superposition of Ca^{2+}-bound nhTMEM16 (PDB: 4WIS; yellow) and Ca^{2+}-bound mTMEM16A structures (PDB: 5OYB; magenta). TMs 4-6 are highlighted in color. **d** Summary of nhTMEM16 and TMEM16A residues that are equivalent to TMEM16F's inner gate residues.

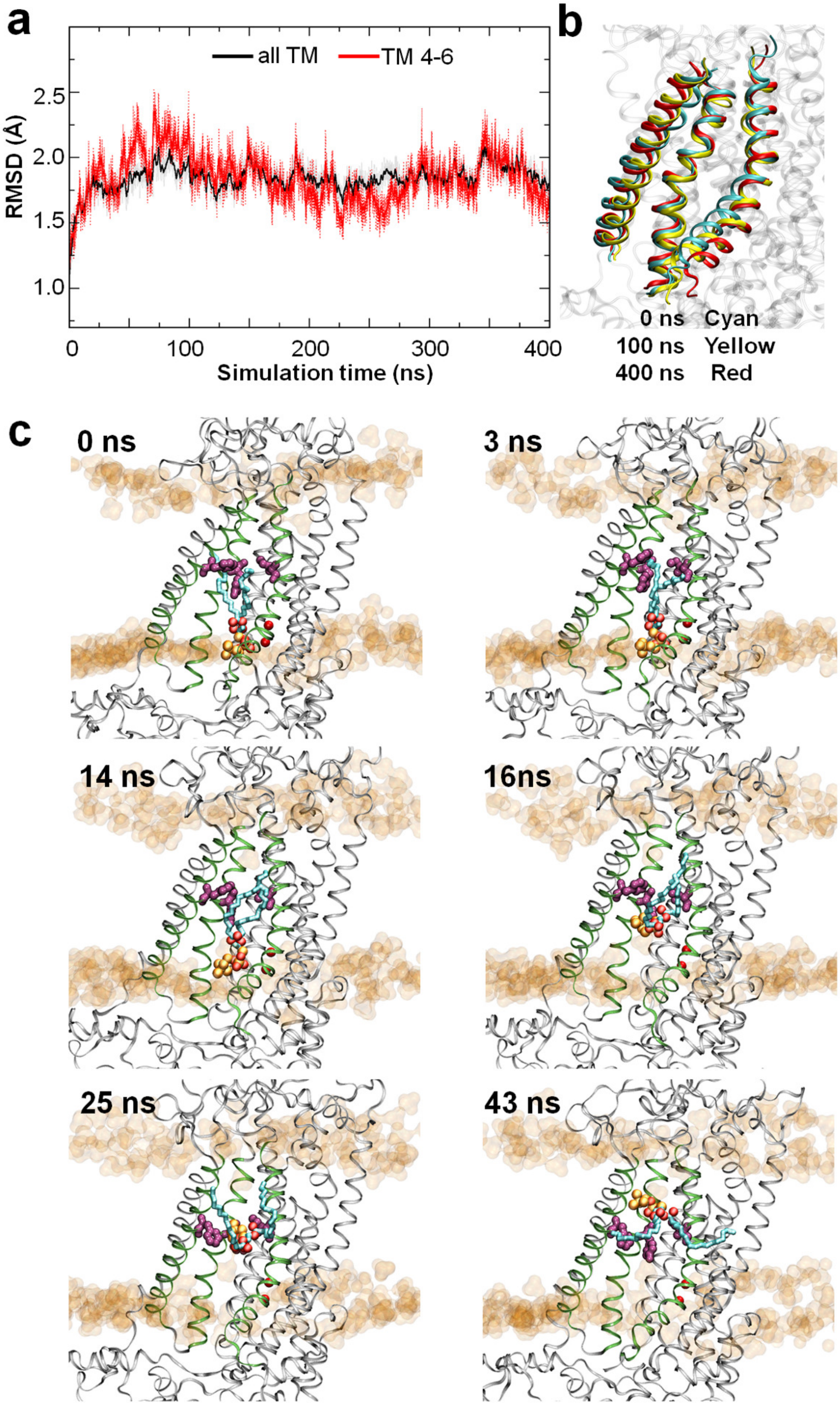
a
all TM
TM 4-6
RMSD (Å)
2.5
2.0
1.5
1.0
0
100
200
300
400
Simulation time (ns)
b
0 ns Cyan
100 ns Yellow
400 ns Red
c
0 ns
3 ns
14 ns
16ns
25 ns
43 ns

Supplementary Figure 2. Molecular dynamic simulation of an open state TMEM16F-CaPLSase homology model. a Atomistic simulations showing the stability of the open state model of TMEM16F. Backbone RMSD values of the entire TM domain (black trace) and TMs 4-6 (red trace) in one of the TMEM16F monomers in a 400-ns simulation with respect to the initial structure. **b** Superposition of the 0 ns (cyan), 100 ns (yellow) and 400 ns (red) structures from the same simulation in a. **c** Atomistic simulation of a POPC phospholipid permeating through the putative inner gate (purple spheres) of TMEM16F. The head group of the POPC molecule (orange spheres with long cyan tails) initially resided in the inner leaflet (0-14 ns), then spontaneously crossed the inner gate region (15-25ns), and eventually reached the outer leaflet of the membrane (43 ns and onward). TMs 4-6 are represented in green cartoon. The phosphate groups of POPC within 15 Å of protein are represented using orange transparent Van der Waals surfaces.

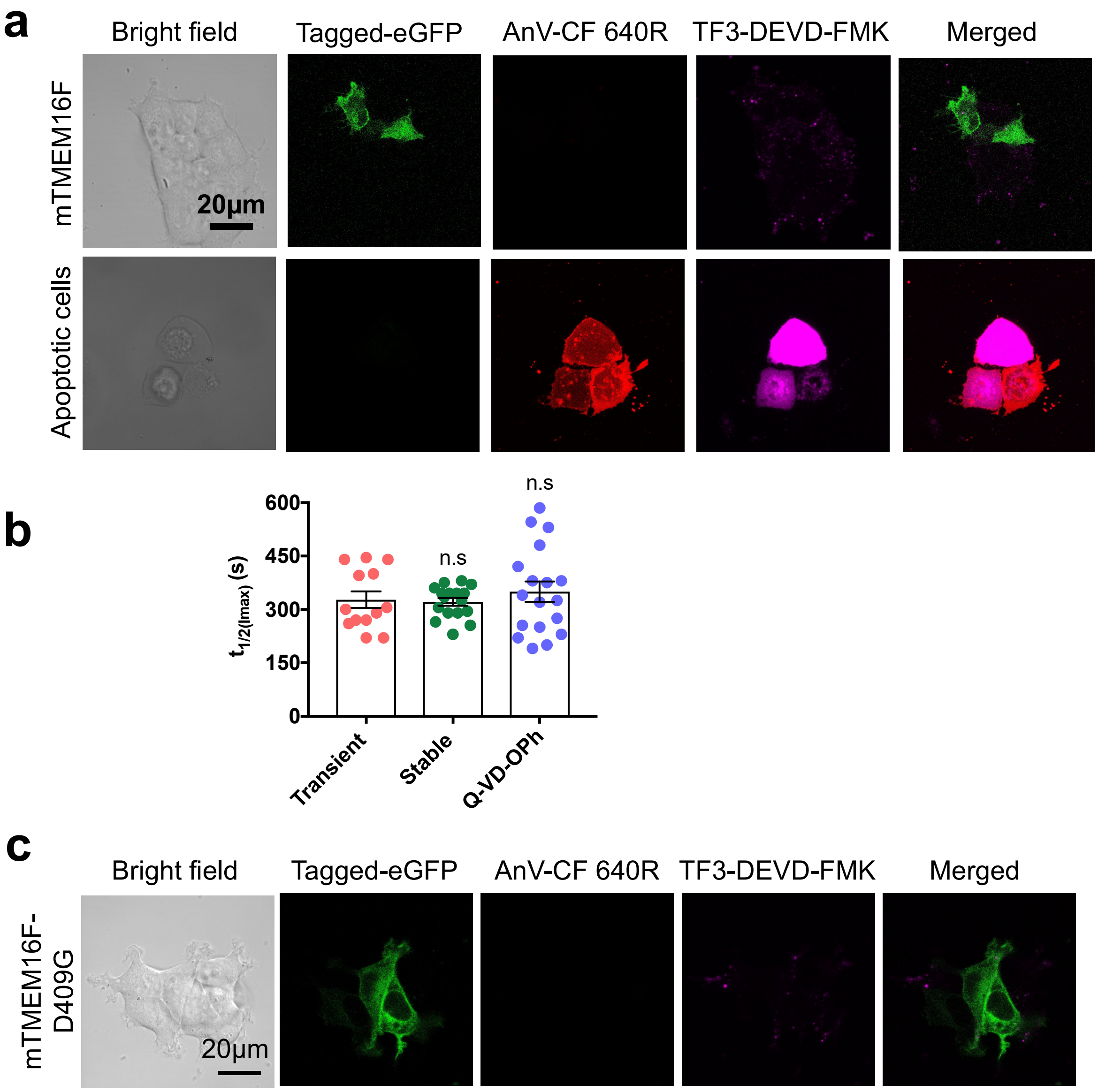

Supplementary Figure 3. Heterologous expression of TMEM16F does not induce spontaneous PS surface exposure and cell apoptosis. a Representative images of TMEM16F-KO HEK293T cells transiently transfected with wildtype mTMEM16F compared with apoptotic cells. mTMEM16F is tagged with eGFP at its C-terminus. PS exposure of apoptotic cells is labeled with AnV-CF 640R (red). Apoptotic cells also show strong cleaved caspases 3/7 activities as labeled with TF3-DEVD-FMK (purple). **b** No difference in scrambling activity for HEK293T cells stably or transiently expressed TMEM16F with and without Q-VD-OPh, a pan-caspase inhibitor. Statistical analysis was performed using One-way ANOVA with Turkey's multiple comparisons test. p-values are 0.98 for Transient vs. Stable and 0.7735 for Transient vs. Q-VD-Oph. n.s denotes not significant. Error bars indicate SEM. Source data are provided as a Source Data file. **c** Representative images of the D409G-expressing cells (green) stained with AnV-CF 640R (red) and TF3-DEVD-FMK (purple). Scale bars, 20 μm.

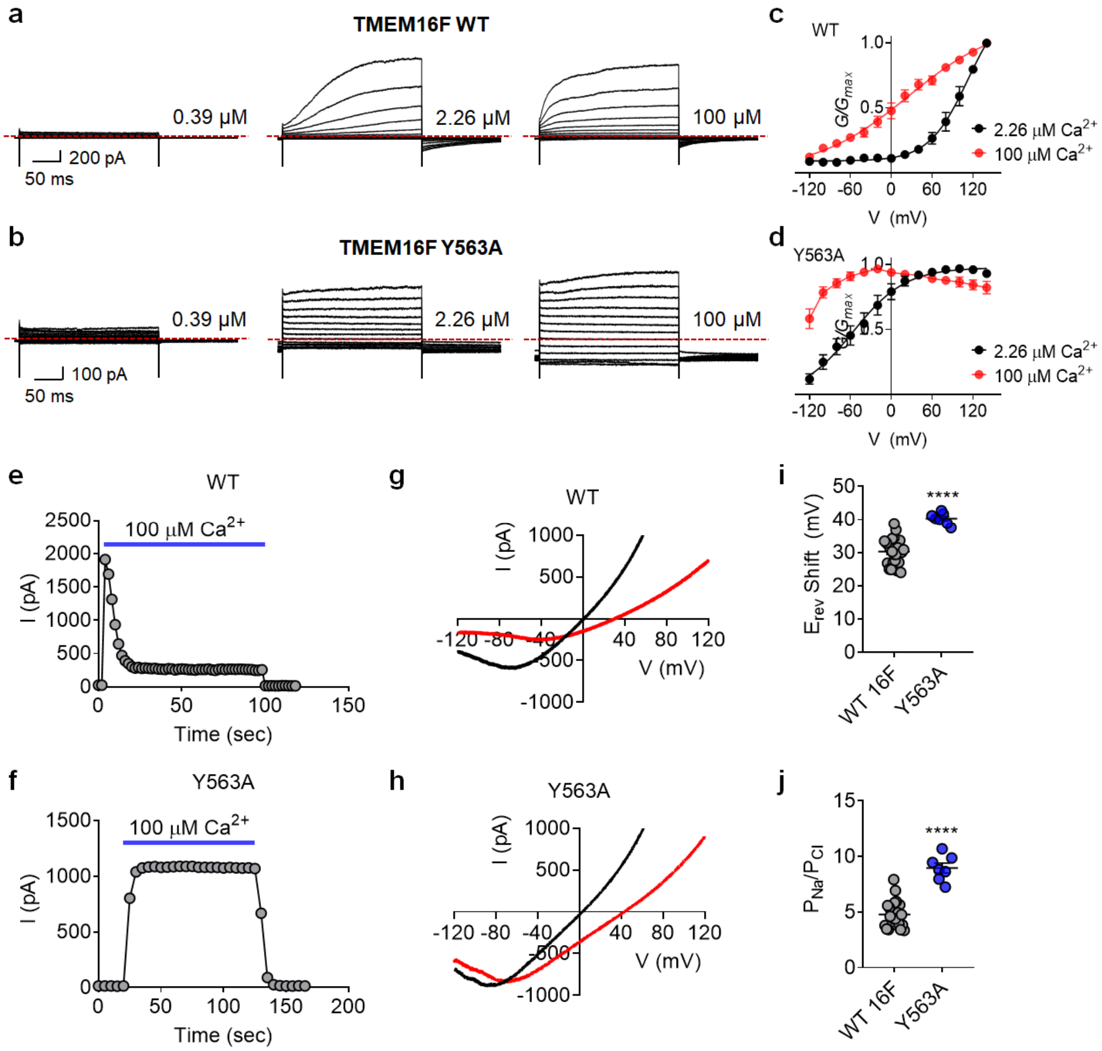

Supplementary Figure 4. Functional characterization of TMEM16F. a, b Representative current traces from inside-out patches excised from HEK293T cells expressing wildtype TMEM16F (WT, **a**) and Y563A-TMEM16F (**b**) when exposed to 0.39, 2.26 and 100 μM Ca^{2+}. Testing potentials were from -120 mV to +140 mV at a 20 mV increment. Both holding and repolarizing potentials were -60 mV. The red dotted lines mark zero current. **c, d** *G-V* relations of WT-TMEM16F (**c**) and Y563A-TMEM16F (d) channels under 2.26 μM and 100 μM Ca^{2+}. Relative conductance was determined by measuring the amplitudes of tail currents measured at the -60 mV repolarization following each test voltage step. Error bars represent SEM. **e, f** Whereas TMEM16F WT exhibited pronounced rundown under 100 μM intracellular Ca^{2+} (e), Y563A abolished channel rundown (f). **g, h** Measurements of the reversal potentials (E_{rev}) for TMEM16F WT (**g**) and Y563A (**h**). Black traces denote currents at symmetric 140 mM NaCl; red traces denote currents upon switching to an intracellular solution with low 14 mM NaCl. An inverted V-shaped voltage ramp ranging from -120 mV to +120 mV was used to elicit channel activation and followed by a reserved +120 mV to -120 mV ramp, which was used to measure reversal potentials. Currents were recorded under 100 μM intracellular Ca^{2+}. **i, j** Changes in the reversal potential (E_{rev}) of TMEM16F WT and Y563A (**i**) and their permeability ratio P_{Na}/P_{Cl} (**j**). Two-tailed un-paired

Student’s t-tests: p-values are both <0.0001 in **i** and **j**. Source data are provided as a Source Data file.

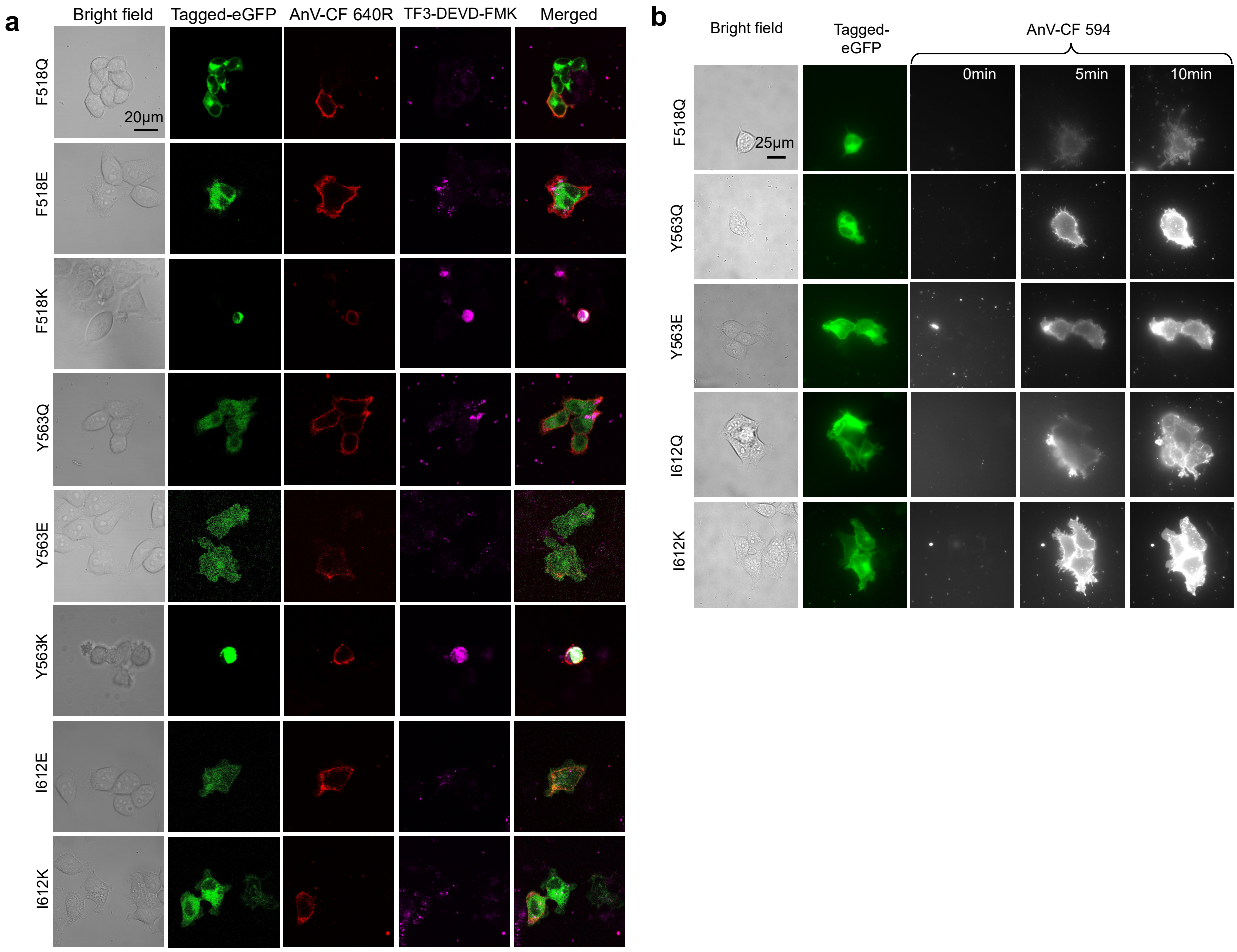

Supplementary Figure 5. Representative images of the TMEM16F inner gate mutations with charged or polar sidechains. a TMEM16F inner gate mutations with charged or polar sidechains are constitutively activated under basal Ca^{2+} level. Images were taken in the absence of ionomycin stimulation. Protein expression of TMEM16F mutants is visualized by their C-terminally tagged eGFP in TMEM16F-KO HEK293T cells. AnV-CF 640R staining labels spontaneously exposed PS on cell surface. Strong and punctuated TF3-DEVD-FMK staining of cleaved caspases 3/7 indicates apoptotic cells. **b** Representative images of ionomycin-induced scrambling activity of TMEM16F-KO HEK293T cells expressing eGFP-tagged TMEM16F- F518Q, Y563Q, Y563E, I612Q and I612K. CF 594-tagged-AnV signal representing phospholipid scrambling was recorded by time-lapsed imaging for 10-minute following application of 5 μM ionomycin (0 min) at a 5-second acquisition interval. All of the experiments based on AnV labeling were done in Ca^{2+}-containing buffer. Scale bars, 20 μm in (**a**) and 25 μm in (**b**).

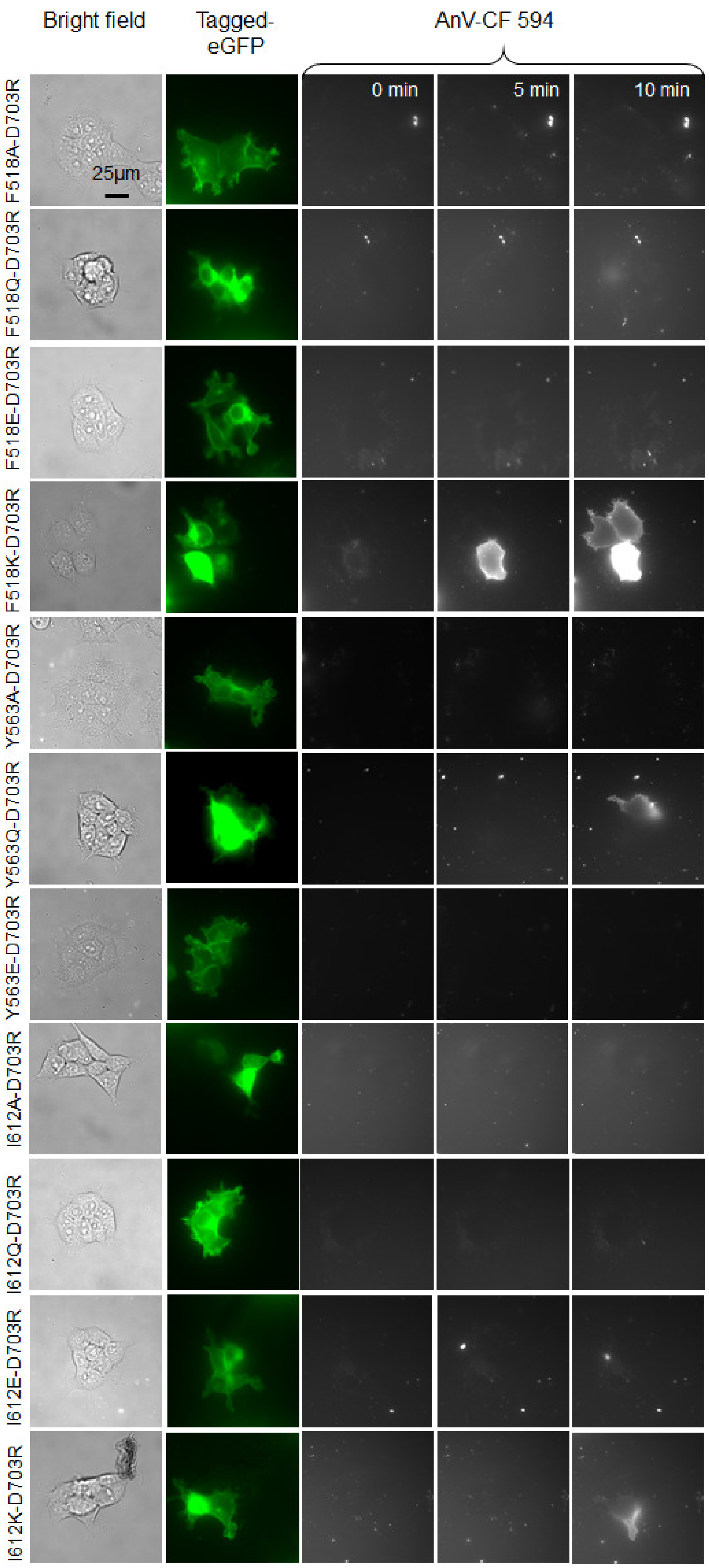

Supplementary Figure 6. Measurement of ionomycin-induced CaPLSase activity of the gain-of-function TMEM16F inner gate mutations coupled with D703R. Representative images of

ionomycin-induced scrambling activity of TMEM16F-KO HEK293T cells expressing eGFP-tagged TMEM16F gain-of-function mutants that are coupled with D703R. CF 594-tagged-AnV signal representing phospholipid scrambling was recorded by time-lapsed imaging for 10-minute following application of 5 μM ionomycin (0 min) at a 5-second acquisition interval. All of the experiments based on AnV labeling were done in Ca^{2+}-containing buffer. Scale bars, 25 μm.

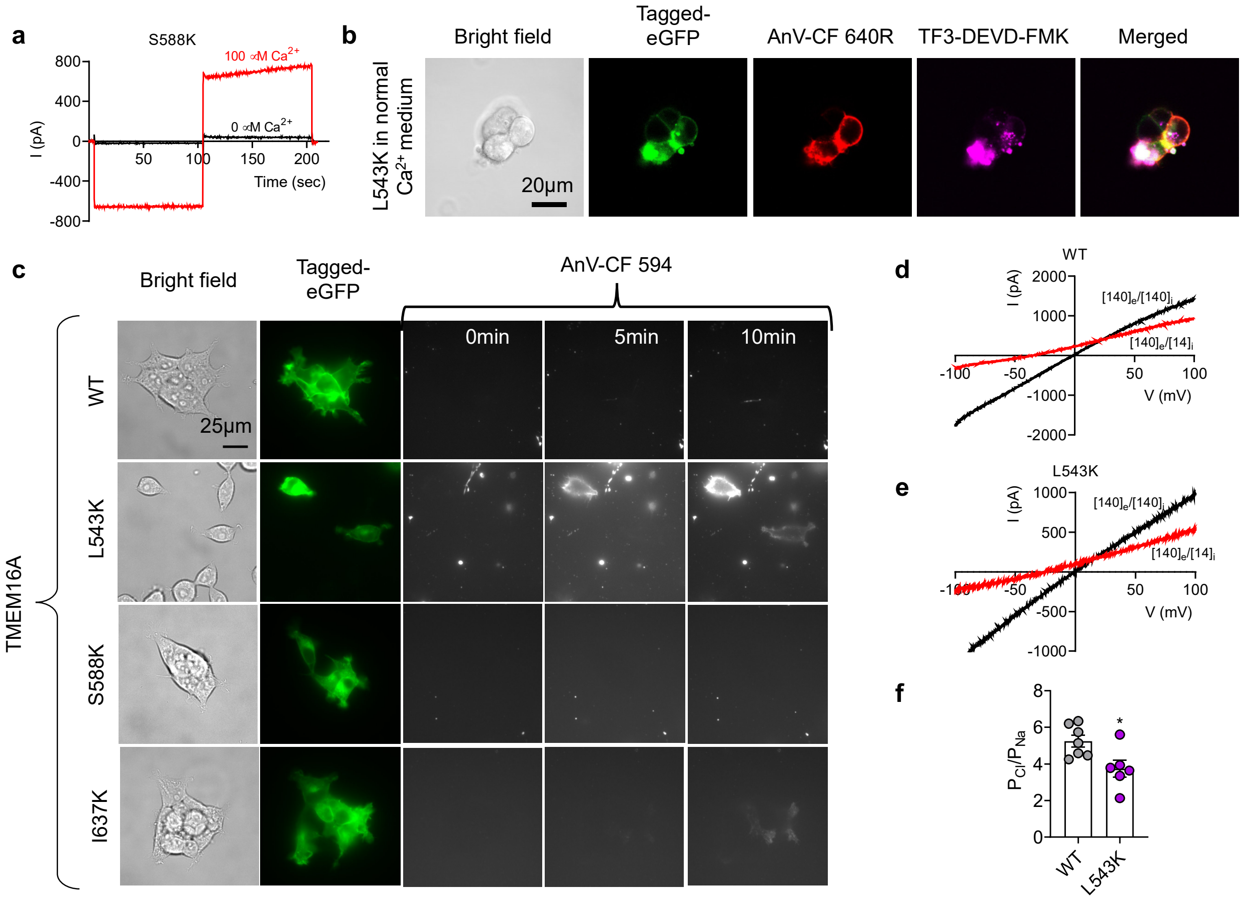

Supplementary Figure 7. Measurement of ionomycin-induced CaPLSase activity of the TMEM16A inner gate mutations. **a** Representative recording showing activation of S588K when 0 µM Ca^{2+} (black trace) or 100 µM Ca^{2+} (red trace) was applied to the cytosolic side of an inside-out patch excised from HEK293T cells expressing TMEM16A S588K. **b** Representative images of TMEM16A L543K-expressing TMEM16F-KO HEK293T cells (green) in normal medium without ionomycin stimulation. AnV-CF 640R (red) labels PS positive cells. TF3-DEVD-FMK staining indicates cleaved caspases 3/7 (purple). **c** Representative images of ionomycin-induced scrambling activity of TMEM16F-KO HEK293T cells expressing eGFP-tagged TMEM16A-WT, -L543K, -S588K and –I637K. CF 594-tagged-AnV signal representing phospholipid scrambling was recorded by time-lapsed imaging for 10-minute post 2.5 µM ionomycin application (0 min) at a 5-second acquisition interval. All of the experiments based on AnV labeling were done in Ca^{2+}-containing buffer. **d, e** Measurements of ion selectivity of TMEM16A WT (**d**), L543K (**e**). Black traces denote currents in symmetric 140 mM NaCl. Red traces denote currents upon switching to an intracellular solution with low 14 mM NaCl. A voltage ramp ranging from -100 mV to +100 mV was used to elicit channel activation. Currents were recorded under 100 µM intracellular Ca^{2+}. **f** The permeability ratios P_{Cl}/P_{Na} of TMEM16A WT and L543K. Two-tailed un-paired Student's t-test: p-value is 0.0191. Scale bars, 20 µm in (**b**) and 25 µm in (**c**). Source data are provided as a Source Data file.

Supplementary Table 1. sgRNA sequence for generating TMEM16F-knockout HEK293T

Target	Species	Cell-line	Target sequence	Genomic location	Exon
TMEM16F	Human	HEK 293T	AATAGTACTCACAAACTCCG	chr12:45302083	2

Supplementary Table 2. Primer sequences used for PCR amplification in Surveyor assay

Target	Test		Sequence
TMEM16F	PCR amplification for Surveyor assay	Forward	TTTTCAGTGGTAGACCTTGCCT
		Reversed	AAGTTCAGCAACCTATTCCCAA
