# Supplementary material for: An inner activation gate controls TMEM16F phospholipid scrambling
Source: Nat Commun. 2019 Apr 23;10:1846. doi: 10.1038/s41467-019-09778-7 (PMC6478717; doi:10.1038/s41467-019-09778-7)
Supplement: Supplementary file 4 — Reporting Summary [file 41467_2019_9778_MOESM4_ESM.pdf]

nature research

Corresponding author(s): Huanghe Yang

Last updated by author(s): Mar 26, 2019

Reporting Summary

Nature Research wishes to improve the reproducibility of the work that we publish. This form provides structure for consistency and transparency in reporting. For further information on Nature Research policies, see Authors & Referees and the Editorial Policy Checklist.

Statistics

For all statistical analyses, confirm that the following items are present in the figure legend, table legend, main text, or Methods section.

n/a	Confirmed	
☐	☒	The exact sample size (*n*) for each experimental group/condition, given as a discrete number and unit of measurement
☐	☒	A statement on whether measurements were taken from distinct samples or whether the same sample was measured repeatedly
☐	☒	The statistical test(s) used AND whether they are one- or two-sided<br>*Only common tests should be described solely by name; describe more complex techniques in the Methods section.*
☒	☐	A description of all covariates tested
☒	☐	A description of any assumptions or corrections, such as tests of normality and adjustment for multiple comparisons
☐	☒	A full description of the statistical parameters including central tendency (e.g. means) or other basic estimates (e.g. regression coefficient) AND variation (e.g. standard deviation) or associated estimates of uncertainty (e.g. confidence intervals)
☐	☒	For null hypothesis testing, the test statistic (e.g. *F*, *t*, *r*) with confidence intervals, effect sizes, degrees of freedom and *P* value noted<br>*Give P values as exact values whenever suitable.*
☒	☐	For Bayesian analysis, information on the choice of priors and Markov chain Monte Carlo settings
☒	☐	For hierarchical and complex designs, identification of the appropriate level for tests and full reporting of outcomes
☒	☐	Estimates of effect sizes (e.g. Cohen's *d*, Pearson's *r*), indicating how they were calculated

Our web collection on statistics for biologists contains articles on many of the points above.

Software and code

Policy information about availability of computer code

Data collection	Meta Fluo (Molecular Devices), Axon PClamp 10 (Molecular Devices), Zeiss-ZEN
Data analysis	Axon Clampfit 10 (Molecular Devices); MATLAB R2018b (Mathworks); Prism 8 (Graphpad); Excel (Microsoft); Pymol (Schrodinger), ImageJ, Zeiss

For manuscripts utilizing custom algorithms or software that are central to the research but not yet described in published literature, software must be made available to editors/reviewers. We strongly encourage code deposition in a community repository (e.g. GitHub). See the Nature Research guidelines for submitting code & software for further information.

Data

Policy information about availability of data

All manuscripts must include a data availability statement. This statement should provide the following information, where applicable:

- Accession codes, unique identifiers, or web links for publicly available datasets
- A list of figures that have associated raw data
- A description of any restrictions on data availability

All data generated or analysed in this study are included in this published article and its supplementary information files. Raw data are available from the corresponding author upon request. Code for quantification of CaPLSase activity using fluorescence microscopy is available at Github (yanghuanghe/scrambling_activitysuch as github).

October 2018

Field-specific reporting

Please select the one below that is the best fit for your research. If you are not sure, read the appropriate sections before making your selection.

☒ Life sciences ☐ Behavioural & social sciences ☐ Ecological, evolutionary & environmental sciences

For a reference copy of the document with all sections, see nature.com/documents/nr-reporting-summary-flat.pdf

Life sciences study design

All studies must disclose on these points even when the disclosure is negative.

Sample size	Most of experiments were repeated at least three times (biological replicates). The sizes were chosen based on past experiences and on the numbers to reach statistical significance.
Data exclusions	Electrophysiology recordings displaying high noises, instability, or current amplitudes too small for analyses were excluded. Out of focus images were excluded from analysis.
Replication	At least three replications were performed and all were successful.
Randomization	No randomization was performed.
Blinding	No blinding was performed.

Reporting for specific materials, systems and methods

We require information from authors about some types of materials, experimental systems and methods used in many studies. Here, indicate whether each material, system or method listed is relevant to your study. If you are not sure if a list item applies to your research, read the appropriate section before selecting a response.

Materials & experimental systems

n/a	Involved in the study	
☒	☐	Antibodies
☐	☒	Eukaryotic cell lines
☒	☐	Palaeontology
☒	☐	Animals and other organisms
☒	☐	Human research participants
☒	☐	Clinical data

Methods

n/a	Involved in the study	
☒	☐	ChIP-seq
☒	☐	Flow cytometry
☒	☐	MRI-based neuroimaging

Eukaryotic cell lines

Policy information about cell lines

Cell line source(s)	HEK293T cells were purchased from Duke Cell Culture Facility; Cas9 control and TMEM16F-KO HEK293T cells were generated by Duke Functional Genomics Facility; HEK293T cell line stably expressing C-terminally tagged eGFP mouse TMEM16F was a gift from Dr. Min Li.
Authentication	HEK293T was authenticated by Duke University Cell Culture Facility.
Mycoplasma contamination	Mycoplasma contamination was checked by Duke University Cell Culture Facility and no further test was performed.
Commonly misidentified lines (See ICLAC register)	N/A
