# Supplementary material for: An inner activation gate controls TMEM16F phospholipid scrambling
Source: Nat Commun. 2019 Apr 23;10:1846. doi: 10.1038/s41467-019-09778-7 (PMC6478717; doi:10.1038/s41467-019-09778-7)
Supplement: Supplementary file 5 — Description of Additional Supplementary Files [file 41467_2019_9778_MOESM5_ESM.doc]

**Title: Movie 1 The steered MD trajectory to build open TMEM16F model.
Description:** The steered MD trajectory where the structure of transmembrane helices 3-6 (yellow) gradually evolved from the closed conformation (green, derived from PDB 5OYB) towards the target open state model (red, derived from PDB 4WIS).

**Title: Movie 2** **Atomistic simulation of a POPC phospholipid permeating through the putative inner gate of TMEM16F.**
**Description:** TMs4-6 are represented in green cartoon. The inner gate residues and Ca^2+^ ions are shown in purple and red, respectively. POPC is represented with cyan acyl chains and yellow and orange head group.
